# Effect of Transplantation of Bone Marrow Stromal Cell- Conditioned Medium on Ovarian Function, Morphology and Cell Death in Cyclophosphamide-Treated Rats

**DOI:** 10.22074/cellj.2018.4919

**Published:** 2018-01-01

**Authors:** Nasrin Khanmohammadi, Hamid Reza Sameni, Moslem Mohammadi, Abbas Pakdel, Majid Mirmohammadkhani, Houman Parsaie, Sam Zarbakhsh

**Affiliations:** 1 Research Center of Nervous System Stem Cells, Faculty of Medicine, Semnan University of Medical Sciences, Semnan, Iran; 2 Molecular and Cell Biology Research Center, Department of Physiology and Pharmacology, School of Medicine, Mazandaran University of Medical Sciences, Sari, Iran; 3 Research Center for Social Determinants of Health Community Medicine Department, Semnan University of Medical Sciences, Semnan, Iran

**Keywords:** Bone Marrow Stromal Cells, Chemotherapy, Conditioned Medium, Ovary, Transplantation

## Abstract

**Objective:**

Although stem cell transplantation has beneficial effects on tissue regeneration, but there are still problems such as high cost and safety issues. Since stem cell therapy is largely dependent on paracrine activity, in this study, utilization of transplantation of bone marrow stromal cells (BMSCs)-secretome instead of the cells, into damaged ovaries was evaluated to overcome the limitations of stem cell transplantation.

**Materials and Methods:**

In this experimental study, BMSCs were cultured and 25-fold concentrated conditioned medium (CM) from BMSCs was prepared. Female rats were injected intraperitoneally with cyclophosphamide (CTX) for 14 days. Then, BMSCs and CM were individually transplanted into bilateral ovaries, and the ovaries were excised after four weeks of treatment. The follicle count was performed using hematoxylin and eosin (H&E) staining and the apoptotic cells were counted using TUNEL assay. Ovarian function was evaluated by monitoring the ability of ovulation and the levels of serum estradiol (E_2_) and follicle-stimulating hormone (FSH).

**Results:**

Evaluation of the ovarian function and structure showed that results of secretome transplantation were almost similar to those of BMSCs transplantation and there was no significant differences between them.

**Conclusion:**

BMSCs-secretome is likely responsible for the therapeutic paracrine effect of BMSCs. Stem cell- secretome is expected to overcome the limitations of stem cell transplantation and become the basis of a novel therapy for ovarian damage.

## Introduction

Chemotherapy is a standard treatment for most forms of malignancies such as breast, cervix and ovaries cancers (1). Despite all the benefits of chemotherapy, it may damage ovaries by destroying primordial oocytes, leading to premature ovarian failure (POF) or early menopause (2). POF is described as secondary infertility induced by alteration in the levels of gonadotropin before the age of 40 (3). Since chemotherapy can increase the risk of sexual dysfunction and infertility (4), it could become a problematic issue in case of girls and young women undergoing chemotherapy (5). Cyclophosphamide (CTX) is one of the most common drugs used in chemotherapy that directly destroys oocytes and stimulates follicular depletion (6, 7). 

Hormone therapy is sometimes used to treat common menopausal problems. But, as hormone therapy mayincrease the risk of cancer or relapse in cancer survivors, so an alternative treatment is required (2). One approach that has recently been noted is stem cell therapy that seemsto be effective in the treatment of infertility in CTX- treated female mice (8, 9). Bone marrow stromal cells(BMSCs) are a type of mesenchymal stem cells that havethe ability to differentiate into other cell lines and may be able to replace damaged cells (10). Moreover, BMSCs canproduce growth factors and cytokines for angiogenesis, mitogenesis and anti-apoptosis such as vascular endothelial growth factor (VEGF), insulin like growth factor 1 (IGF1), basic fibroblast growth factor (bFGF) and hepatocytegrowth factor (HGF) (11-13). Some studies showed BMSC transplantation could repair damaged ovary in rats that were undergoing chemotherapy (11, 13).

Despite numerous benefits of stem cell therapy, it has its potential restrict (10). Moreover, BMSCs can produce growth factors and cytokines for angiogenesis, mitogenesis and anti-apoptosis factors such as ions, immunological rejection may still occur and there is the possibility of malignant transformation (14). An alternative method is to use the secretome instead of the stem cells themselves. This idea is based on the concept that the secretome is responsible for a considerable proportion of the therapeutic potential of stem cells (15, 16). 

In this study, ovaries were damaged by CTX treatment in rats. Then, BMSCs and conditioned medium (CM) of the cells were directly injected into the ovaries to compare the effect of transplantation of BMSCs and their CM on function and structure of damaged ovaries.

## Materials and Methods

In this experimental study, 40 adult female Wistar rats (180-200 g) were kept under controlled temperature (27 ± 2°C) with free access to food and water. Vaginal smear was prepared daily and only those displaying at least two consecutive normal estrus cycles, were included in the experiments. All animal protocols were approved by the Research Council of Semnan University of Medical Sciences, Semnan, Iran. 

## Bone marrow stromal cells culture

BMSCs were prepared from an adult female rat. After killing the rat, femurs and tibias were dissected out. The bone marrow was ejected with 10 ml of Dulbecco’s Modified Eagle Medium (DMEM, Gibco, Germany), cultured in DMEM containing 10% fetal bovine serum (FBS, Gibco, Germany) and 1% penicillin/streptomycin (Gibco, Germany), and incubated at 37°C with 95% humidity and 5% CO_2_. Two days later, the culture medium was replaced with fresh medium to remove debris (17).

## Analysis of the cell surface antigen markers 

To analyze BMSCs surface antigen markers, flow cytometry was performed. At least 1×10^5^ cells were incubated with fluorescence-labeled monoclonal antibodies against CD29, CD34, CD44, CD45 and CD90 (Sigma, USA). After 10 minutes of rinsing with PBS, expression of the CD markers in BMSCs were analyzed by flow cytometry (BD FACS Calibur) (11).

## Preparation of conditioned medium

BMSCs that reached more than 70% confluence, were re-fed with serum-free DMEM. After culturing 1×10^6^ BMSCs for 24 hours, the CM was collected and concentrated 25-fold using ultrafiltration units with a 5 kDa molecular weight cut-off (Amicon, Millipore, USA). The concentrated medium was stored at -80°C for future use (18, 19). 

## Creating the chemotherapy model 

The POF model of chemotherapy was created according to the method described by Takehara et al. (20) . For this purpose, initially CTX (Sigma, china) diluted in normal saline was intraperitoneally injected (50 mg/kg). Then, CTX 8 mg/kg/day was injected for 13 consecutive days (a total of 14 doses). 

## Procedure of transplantation

The rats were randomly divided into four groups as follow (n=10 in each group): i. Normal group: received no treatment, ii. Control group: after induction of POF, 20 µl of culture medium was directly injected into the bilateral ovaries, iii. BMSCs group: after induction of POF, 2×10^6^ BMSCs suspended in 20 µl of culture medium were directly injected into the bilateral ovaries, and iv. CM group: after induction of POF, 20 µl of the CM was directly injected into the bilateral ovaries (2).

## Tracking of transplanted bone marrow stromal cells in the ovaries

BMSCs were labeled with DiI (1,1`-dioctadecyl3,^3^,3`, 3`-tetramethylindocarbocyanine perchlorate, Sigma, China) in the ovaries to show the presence and viability of the transplanted cells after four weeks. Briefly, after suspending the cells, 5 µl/ml DiI was added into the culture medium and incubated for 20 minutes. Then, the cells were centrifuged, rinsed with PBS, and suspended again for transplantation. Four weeks after transplantation, prepared paraffin sections and the labeled cells were detected by fluorescence microscopy (Motic, AE31, Spain) (21, 22).

## Hormonal examination

Four weeks after transplantation, levels of serum estradiol (E_2_) and follicle-stimulating hormone (FSH) were measured by enzyme-linked immunosorbent assay (ELISA) kits (East bio pharm, China) for rats, according to the manufacturer’s instructions (11, 23-25).

## Assess the ability of ovulation

Four weeks after transplantation, the rats were super ovulated by an intraperitoneal injection of 150 IU/kg of pregnant mare serum gonadotropin (PMSG, Sigma, china), followed by an intraperitoneal injection of 75 IU/kg of human chorionic gonadotropin (hCG, Sigma, china) administered 48 hours later (26). The oocytes were obtained from the ampulla portion of the oviduct, 14-16 hours after hCG injection (2).

## Apoptosis detection

Four weeks after transplantation, apoptotic granulosa cells (GCs) were assessed by TUNEL assay kit (Roche, Germany). Briefly, the sections were treated with 20 g/ml proteinase K for 10 minutes and 0.1% Triton X-100 in 0.1% sodium citrate for 2 minutes on ice. The sections were placed in the TUNEL reaction mixture and stained using diaminobenzidine (DAB) solution for 10 minutes at room temperature. Then, they were counter-stained using hematoxylin. At least 100 GCs were counted in eight random fields under a fluorescence microscope (Motic, AE31, Spain) to calculate the percentage of apoptotic cells (11, 27-29). 

## Ovarian follicle counts

Ovarian follicles were counted according to the method described by Sun et al. (2). Briefly, four weeks after transplantation, the ovaries were collected, fixed in paraformaldehyde, dehydrated, paraffin-embedded and serially sectioned with 5-µm thicknesses. Five representative sections from each ovary were randomly chosen and routine hematoxylin and eosin (H&E) staining was performed. Finally, the number of primordial, primary, secondary and antral follicles was evaluated.

## Statistical analyses

All data were analyzed by one-way analysis of variance (ANOVA) followed by the Tukey’s post-test. Obtained data were presented as mean ± SD, and a P<0.05 was considered statistically significant.

## Results

### Bone marrow stromal cells culture and characterization

**Fig.1:**
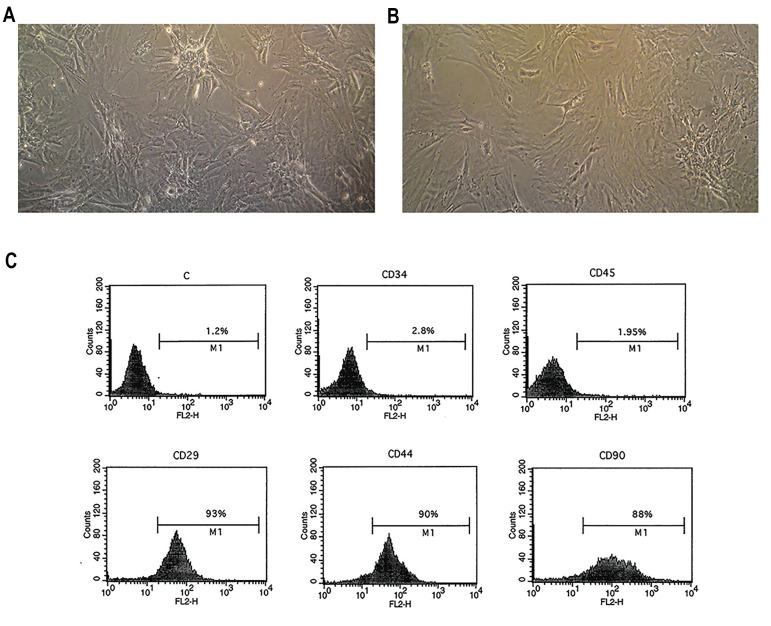
The isolation and identification of bone marrow stromal cells (BMSCs). Cultured BMSCs at A. Passages 1, B. 3 (×100), and C. Flow cytometry results showing BMSCs positive for CD29, CD44 and CD90, and negative for CD34 and CD45.

In the early days of BMSCs culture, the cells were spindle-shaped and formed colonies. After a few days, the morphology of the BMSCs was similar to that of fibroblast and after repeating passages, the morphology of the cells became homogeneous (Fig .1A, B). Most of the cells were immunopositive for markers of mesenchymal stromal stem cells namely, CD29, CD44 and CD90, and were immunonegative for hematopoietic markers namely, CD34 and CD45 (Fig .1C). 

### Identification of bone marrow stromal cells in the ovaries 

**Fig.2:**
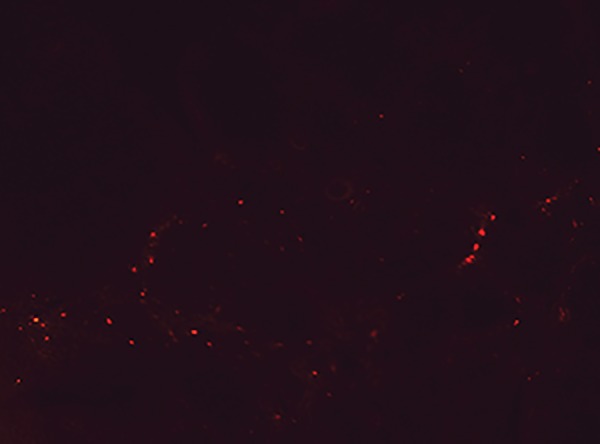
Bone marrow stromal cells labeled with DiI (appeared as red spots) in an ovary section, four weeks after transplantation (×100).

Histochemistry technique showed that the transplanted BMSCs that were labeled with DiI appeared as red spots in the sections of ovaries. The results confirmed the presence and viability of the transplanted cells in the ovaries, four weeks after transplantation (Fig .2). 

### Levels of estradiol and follicle-stimulating hormone

**Fig.3:**
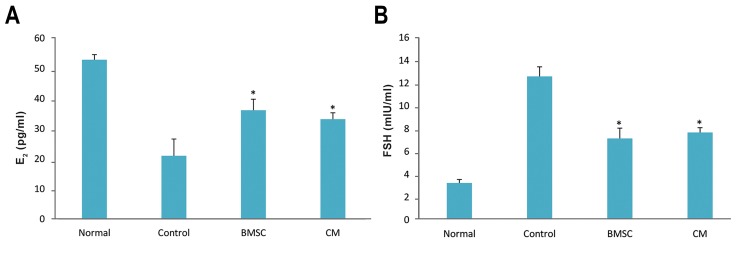
Levels of ovarian hormones after bone marrow stromal cell (BMSC) transplantation. Four weeks after transplantation, A. Serum levels of estradiol (E_2_) in BMSCs and conditioned medium (CM) groups were significantly higher than control group and B. While serum levels of follicle-stimulating hormone (FSH) in BMSCs and CM groups were significantly lower than control group. *; P<0.05 vs. control group.

The levels of serum E2 in the BMSCs and CM groups were significantly higher than those of the control group; however, the levels of serum FSH in the BMSCs and CM groups were significantly lower than those of the control group. No statistically significant differences were observed between the BMSCs and CM groups (P<0.05, Fig .3). 

### The ability of ovulation

**Fig.4:**
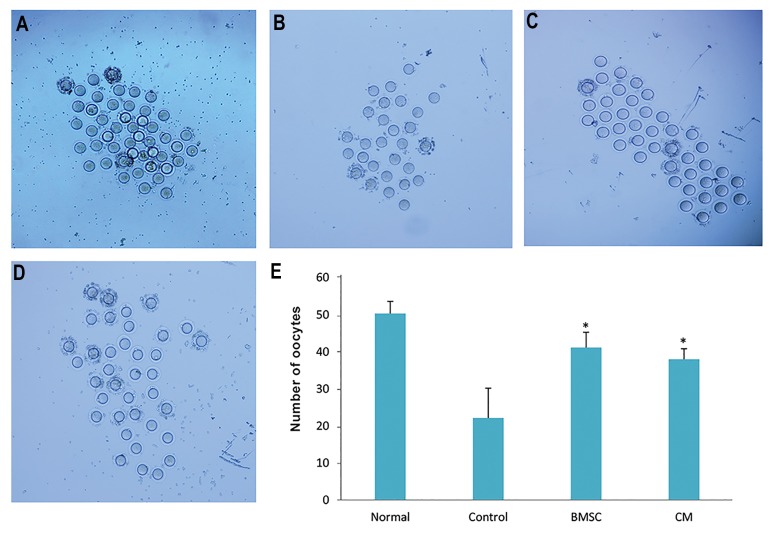
The number of oocytes after bone marrow stromal cell (BMSC) transplantation. Collected oocytes after super ovulation in A. Normal, B. Control, C. BMSCs, D. Conditioned medium (CM) groups (×40), and E. The number of oocytes after super ovulation in all groups. *; P<0.05 vs. control group.

The results of the ability of ovulation showed that the number of oocytes in the BMSCs and CM groups was significantly greater than the control group, but there was no statistically significant differences between the BMSCs and CM groups (P<0.05, Fig .4).

### Apoptosis of granulosa cells

**Fig.5:**
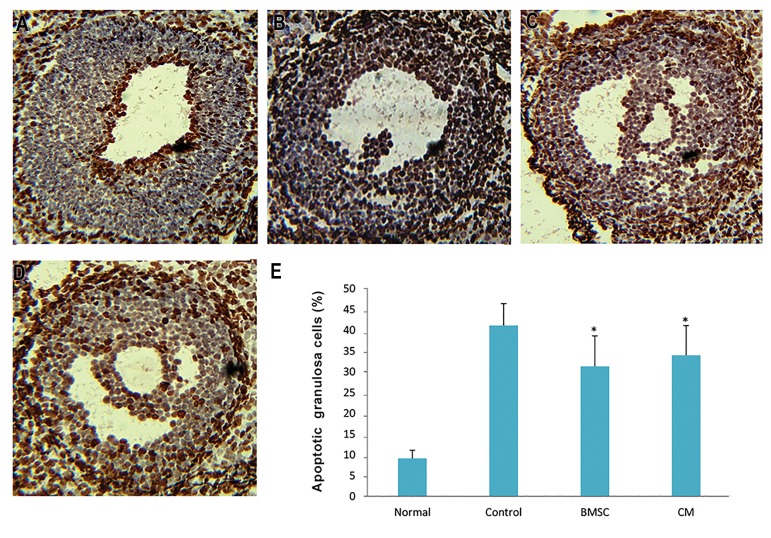
The apoptosis of ovaries after bone marrow stromal cell (BMSC) transplantation. Apoptotic granulosa cells (GCs) are marked in brown using TUNEL staining in A. Normal, B. Control, C. BMSCs, D. Conditioned medium (CM) groups (×200), and E. The number of TUNEL-positive GCs in all groups. *; P<0.05 vs. control group.

The percentage of TUNEL-positive GCs in the ovaries of the BMSCs and CM groups was significantly lower than that of the control group, but no statistically significant differences were observed between the BMSCs and CM groups (P<0.05, Fig .5). 

### Ovarian follicle counts

**Fig.6:**
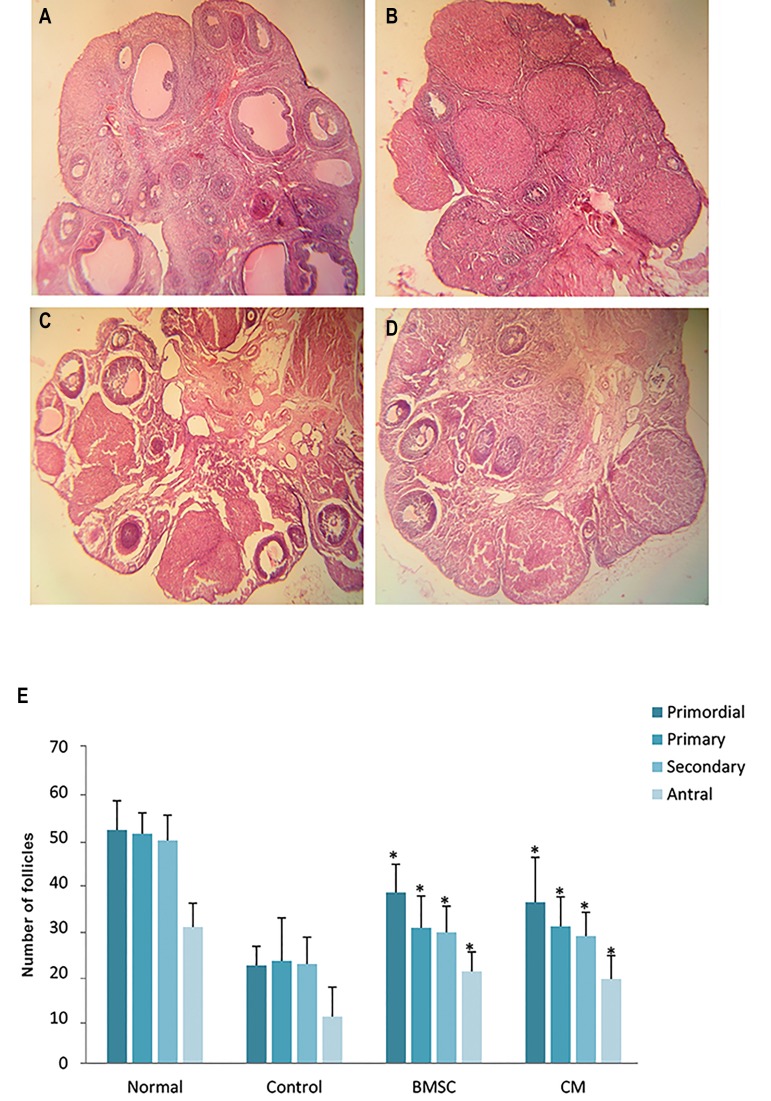
The number of ovarian follicles after bone marrow stromal cell (BMSC) transplantation. Hematoxylin and eosin (H&E) staining of ovaries in A. Normal, B. Control, C. BMSCs, D. Conditioned medium (CM) groups (×40), and E. The number of follicles at different stages in all groups. *; P<0.05 versus control group.

In the BMSCs and CM groups, H&E staining indicated that the number of follicles at different stages was significantly higher than that of the control group. There was no statistically significant differences in the number of follicles between the BMSCs and CM groups (P<0.05, Fig .6).

## Discussion

Following chemotherapy, the ovaries may be this study, for the first time, we compared the effect damaged as reflected by follicle loss, cortical fibrosis, of BMSCs transplantation and that of secretome and vascular damage (30, 31). Some articles have transplantation by evaluating the improvements of shown that BMSCs transplantation after chemotherapy ovarian function and structure in a chemotherapy induced POF rat model. Overall, the results of secretome transplantation were almost similar to those of BMSCs transplantation and there was no significant differences between them.

It has been suggested that the paracrine activity of growth factors and cytokines released by transplanted stem cells may account for their therapeutic potential (14, 15). Some articles have reported that beneficial effect of stem cells on degenerative diseases, is due to their ability to secrete trophic factors that have beneficial impact on damaged tissue, rather than their ability to differentiate into the needed cells (32, 33). Different studies on the factors secreted by stem cells have shown that these factors, in the absence of stem cells, may regenerate tissues under several conditions (34, 35). Secreted factors are referred to as secretome that are released in the medium where the stem cells are cultured, so, the medium is called CM (35).

We cultured BMSCs and obtained the CM. Next, BMSCs and the CM were individually transplanted into rat ovaries after chemotherapy. BMSCs expressed CD29, CD44 and CD90, but not CD34 and CD45, which was in agreement with other reports (11, 17). Since DiI labeling is a simple and stable technique which persists for a long time to trace cells in *in vivo* experiments (36), we labeled BMSCs with DiI and transplanted them into the ovaries. Moreover, it was shown that the transplanted BMSCs could survive in the ovaries after four weeks. This result was in agreement with those of other studies (21, 22). The results of histological, hormonal and functional assessments including counting the number of follicles, apoptotic cells and oocytes, and measuring serum levels of E2 and FSH, showed that transplantation of BMSCs and their CM were significantly more effective in repairing the ovaries as compared to control group. The results of BMSCs transplantation group were consistent with other reports (11, 13) which showed that BMSCs transplantation into damaged ovaries could repair them. However, the effect of transplantation of BMSCs-secretome on damaged ovaries following chemotherapy, has not been previously investigated. 

BMSCs are emerging as strong candidates for cell therapy in the ovaries because they produce growth factors such as VEGF, IGF-1, HGF and bFGF that can prevent cell apoptosis and promote functional recovery (11-13, 37). VEGF is an angiogenic cytokine that promotes formation of new capillary networks providing nutrition for GCs (11, 13, 37). IGF-1 is a growth hormone that stimulates GC proliferation by regulating DNA replication of theca cells and GCs. IGF-1 enhances the function of gonadotropin hormones, regulates aromatase activity, promotes follicular antrum formation and inhibits apoptosis (11, 13). HGF is a cytokine that promotes follicle maturation and suppresses apoptosis in ovarian follicles and GCs (11). Another growth factor is bFGF which acts as an initiator of folliculogenesis by inducing primordial follicle development (37). Some articles have reported that these cytokines and growth factors are secreted by the stem cells into their CM (34, 38). Despite the benefits of stem cells, the use of secretome-containing CM has many advantages over the use of stem cells, as CM can be packaged, manufactured, freeze-dried and transported more easily, and there is no need for donor-recipient matching to avoid rejection problems (34). Moreover, the most serious concern about stem cells is the possibility of malignant transformation (39). In relation to this topic, Lee et al. (35) have shown that repairing liver tissue with CM transplantation of adipose-derived stem cells is comparable to adipose- derived stem cell transplantation. 

Since the results of transplantation of BMSCs and their CM were almost similar, cell-free therapy using secretome can probably be a suitable way to overcome the limitations of stem cell-based therapy. Since paracrine factors produced by stem cells can accumulate in the CM, it can be used as a cell free- therapy. Mesenchymal stem cell secretome contains a large number of cytokines and growth factors that are critical for repairing damaged tissues (34, 35). More research is necessary to clarify the molecular mechanisms through which stem cell-CM repairs the ovaries.

## Conclusion

BMSCs and BMSCs-secretome produced almost similar results in terms of ovarian regeneration in a chemotherapy-induced POF model in rats. These results show BMSCs-secretome is likely responsible for the therapeutic paracrine effect of BMSCs. Stem cell-secretome is expected to overcome the limitations of stem cell transplantation and become the basis of a novel therapy for ovarian damage. 
